# High-fat diet may increase the risk of insulin resistance by inducing dysbiosis

**DOI:** 10.1016/j.metop.2025.100381

**Published:** 2025-07-22

**Authors:** Ebrahim Abbasi, Iraj Khodadadi

**Affiliations:** Nutrition Health Research Center, Hamadan University of Medical Sciences, Hamadan, Iran

**Keywords:** Diabetes mellitus, Insulin resistance, High fat diet, Gut microbiome

## Abstract

High-fat diet (HFD) poses various health risks, such as obesity, insulin resistance (IR), fatty liver, gut microbiota dysbiosis, cognitive impairment, inflammation, and oxidative stress. HFD can alter gastrointestinal function and structure, resulting in changes of the intestinal mucosa, gastric secretions, intestinal connective tissue, intestinal motility, intestinal metabolomics profiles, and intestinal microbiota. The intestine and its microbiota process nutrients and produce molecules that can regulate insulin action and secretion. Changes in the gut microbiome (dysbiosis) and their products may have long-term effects that are not fully understood. Gut microbiota have long been documented to induce metabolic endotoxemia by releasing lipopolysaccharide, which causes systemic inflammation and insulin resistance (IR). HFD may has direct roles in the development of insulin resistance (IR). HFD can induce dysbiosis by reducing SCFAs and decreasing the activation of free fatty acid receptors (FFARs). Furthermore, HFD can increase the activation of the toll-like receptor (TLR) pathway. Hence, HFD by inducing inflammation, oxidative stress, endotoxemia, and hyperglycemia can increase the risk of IR. Therefore, this review aims to delineate the role of gut microbiota directly or indirectly involved in HFD-induced IR. These findings may clarify valuable preventive and therapeutic targets for countermeasures to IR in people who use the Western diet.

## 1 Introduction

The gastrointestinal tract is the largest boundary between the environment and the host. The intestine acts as a barrier system for protecting the host. Hence, it prevents toxins, bacteria, and hazardous substances from entering the bloodstream and tissues. The intestinal tract by various protective mechanisms, including tight junctions, gut microbiome, and mucus layer prevent endotoxemia [1].

It is recognized that the Western diet (the typical American diet) has about 36–40 % fat, which provides 50–60 % of the required energy. The Western diet, often described as low fiber and high fat content, is typically related to an increased risk of health issues [2]. Previous studies have reported that most patients with chronic disease had a history of specific dietary misbalances, including excessive sugar consumption and/or HFD intake. Hence, excessive sugar consumption and HFD should be treated as risk factors for chronic diseases [3].

**Fig. 1:**
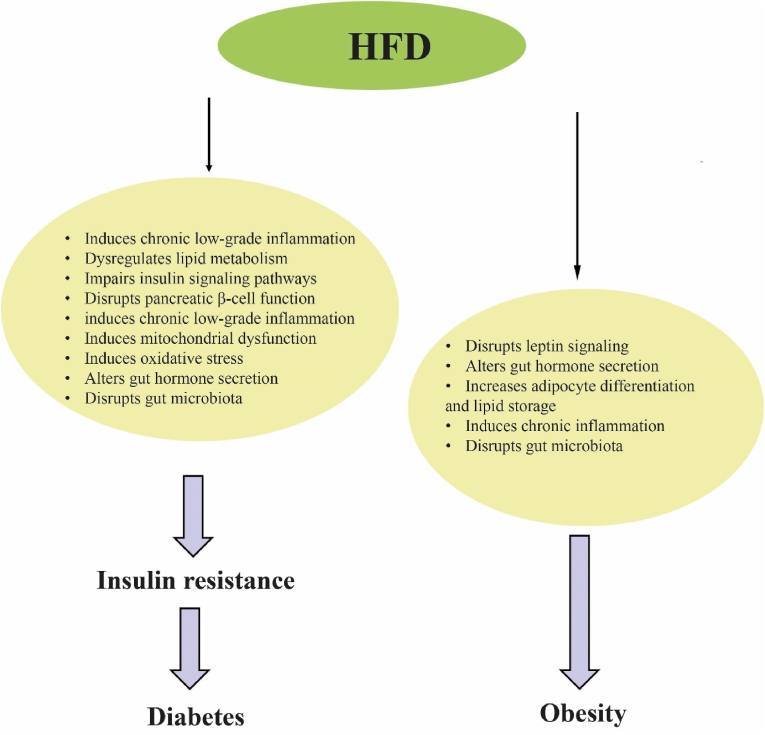
Health concerns that may be induced by HFD or Western diet [[3], [4], [5]].

HFD may induces obesity, fatty liver, insulin resistance (IR), intestinal injury, gut microbiota dysbiosis, cognitive impairment, inflammation, and oxidative stress (Fig. 1) [[3], [4], [5]]. HFD can change the composition of the gut microbiota and reduce its diversity [[6], [7], [8]]. Dysbiosis (imbalances in gut microbiota) has been related to irritable bowel syndrome, diabetes, fatty liver, and different types of cancer [9].

## 2 Methods

The words insulin resistance, high-fat diet, gut microbiome, and dysbiosis were entered into databases, such as Scopus, ScienceDirect, Google Scholar, and PubMed.

Inclusion criteria: English-language original or review, and meta-analyses papers published previously. Different diets, including high-fat, high-fiber, and Mediterranean, which affect the gut microbiota and then induce insulin resistance, were investigated. The effects of probiotics, or prebiotics, on insulin resistance and gut microbiota were investigated. Furthermore, changes of insulin sensitivity, microbial diversity, or biochemical markers (e.g., fasting glucose, inflammatory factors, HbA1c, etc.) in the HFD model were investigated. Exclusion criteria: Non-English papers and non-peer-reviewed studies (e.g., editorial papers, conference abstracts). The experiments focus only on obesity, HFD, without evaluating insulin resistance or gut microbiota.

## 3 Results and discussion

### 3.1 HFD and risk of insulin resistance (IR)

IR was described in 1936 as a metabolic disorder characterized by reduced cellular sensitivity to insulin in adipose tissue, skeletal muscle, and liver [10]. IR has a key role in the dysfunction of pancreatic β-cells and the development and progression of type 2 diabetes (T2D) [11]. IR can lead to oxidative stress, endothelial dysfunction, and inflammatory response and is strongly related to other metabolic risk factors such as hyperlipidemia, obesity, and hypertension [12]. IR is a multifactorial disorder and emerges several years before the onset of diabetes [13]. IR is also related to cardiovascular diseases and cancer [14,15]. An analysis of the National Health and Nutrition Examination Survey (NHANES) showed that 40 % of USA adults aged 18 to 44 have IR [16].

Inflammation, hyperglycemia, oxidative stress, and abnormal lipid levels, as seen in HFD-fed models [17]. Since IR is well recognized as one of the main causes of T2D and as a cardiovascular disease (CVD) risk factor, it is important to distinguish the associated risk factors, related mechanisms, and physiological changes in HFD-fed subjects [13]. One mechanism is that HFD leads to oxidative stress and inflammation, which reduces glucose utilization. HFD can reduce adiponectin levels and consequently affect glucose metabolism [18]. Furthermore, several alterations in the gastrointestinal system, including abnormalities in intestinal structure and function, and microbial composition, have been reported in HFD-fed models. These abnormalities in the gut microbiota and intestinal structure in HFD-fed subjects may contribute to the development of diabetes.

### 3.2 HFD changes the intestinal structure and function

In the gastrointestinal tract, microorganisms are recognized as the first line of defense against various pathogens and can influence different physiological, biochemical, and immunological functions. Moreover, the gut microflora may affect the homeostasis of plasma proteins, hormones, and lipids. In healthy subjects, more than 100 trillion useful bacterial cells of many species colonize the gut, constituting the intestinal microbiota [19].

Certain commensal microbes synthesize growth factors and vitamins and together may create a favorable physiological environment [20,21]. Gut microbiota can produce vitamin K and B12, biotin, folate, thiamine, pyridoxine, and nicotinic acid [22]. The gastrointestinal flora benefits the human body in numerous ways. The gut microbiome involves lipid, carbohydrate, and protein digestion, and provides the main capacities for the fermentation of dietary fibres. Furthermore, dysbiosis of gut microbiota can lead to immune dysregulation, changed energy regulation, and gut hormone regulation [23]. Disruption of gut microbiota may be related to many diseases, such as inflammatory bowel disease, gastrointestinal cancers, obesity, IR, diabetes, autism, and allergy [24,25].

Findings obtained from various studies have shown a causal association between the gut microbiota and IR [24]. In the last decade, scientists have been dramatically interested in using gut microbiota as a target for the prevention of IR, liver diseases, and T2D. In diabetic patients, the release of gut hormones [e.g., glucagon-like peptide (GLP)] is reduced due to a changed gut microbiota [26,27] since gut microorganisms affect the plasma metabolome and are related to IR [28].

**Fig. 2:**
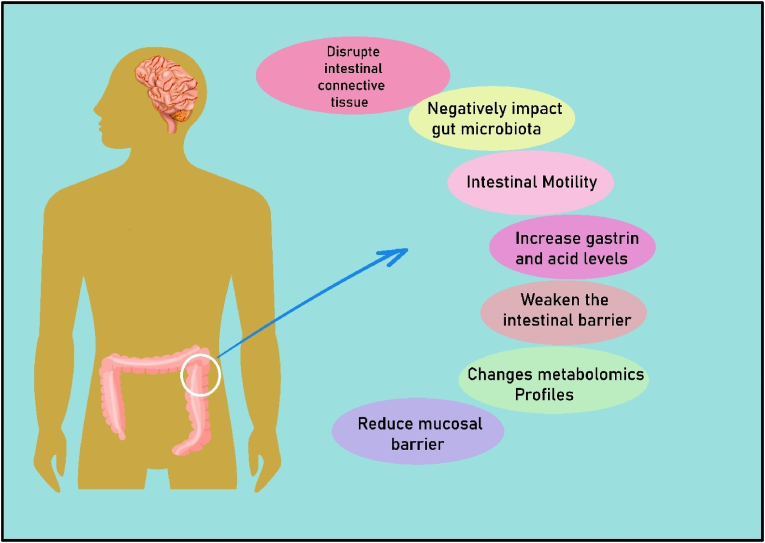
High-fat diet (HFD) can lead to intestinal alterations [1,[30], [31], [32], [33]].

HFD can alter the intestinal mucosa [29], gastric secretions, intestinal connective tissue, intestinal motility, intestinal metabolomics profiles, and intestinal microbiota [1,[30], [31], [32], [33]] (Fig. 2). The gastrointestinal mucosal cells are damaged by HFD because of the decreased anti-apoptotic and increased pro-apoptotic proteins [30]. Previous studies also showed that HFD disrupts the crypt structure and goblet cells in the colon, reduces the regeneration rate of colonic epithelial cells, and increases intestinal inflammatory markers [30]. In another experiment, Lee et al., showed that the numbers of intestinal goblet cells were significantly reduced by HFD in mice. Moreover, animals that received HFD showed pathological alterations in the large intestine epithelium, were more susceptible to experimental colitis, and had severe colonic inflammation along with an increased population of *Proteobacteria* and *Atopobium* spp [34].

### 3.3 Role of the intestine in the prevalence of IR

It has been established that the intestine controls blood glucose and peripheral insulin levels. The intestine regulates glucose absorption and barrier integrity, both of which play a crucial role in the development of IR through various hormones such as GLP and gut peptide YY. GLP-1 lowers insulin clearance and regulates blood glucose levels [26,35,36]. Intestinal microbes also regulate carbohydrate metabolism and participate in an integrated response that is affected by environmental and genetic factors, which change insulin secretion and blood glucose levels [37].

In animal models, alterations in intestinal barrier integrity, intestinal hormonal secretions, and lipid and glucose metabolisms can induce IR. Furthermore, lipopolysaccharide (LPS), which is produced by pathogenic bacteria, activates the toll-like receptor-4 (TLR-4) axis, and consequently, induces low-grade inflammation and changes in energy homeostasis, contributing to the prevalence of IR [38]. It has been reported that T2D and obesity are accompanied by elevated penetration of LPS from gut bacteria into blood with subsequent low-grade inflammation [39]. Therefore, a change in intestinal permeability, inflammation, oxidative stress, as well as changes in the gut microbiota and lipid homeostasis, are the contributing factors to the prevalence of IR [40].

Regarding the importance of the intestine in the prevalence of IR, it was suggested that T2D may start in the intestine [41]. For example, a high-fat and/or high-carbohydrate diet induces IR by both gut microbiota-dependent and independent mechanisms [42]. Through induction of intestinal barrier permeability, a high-fat diet can change the gut microbiota and increase interferon-gamma (IFNγ) and interleukin-1 beta (IL-1β) levels, and consequently induce IR [40].

### 3.4 HFD induces inflammation and oxidative stress

HFD can damage the proteins, membrane lipids, and DNA by elevating oxidative stress, and may permanently affect the genetic code of tissues [43]. Elevated reactive oxygen species (ROS) levels can cause cellular damage and induce inflammation in the intestine [43]. Gastrointestinal inflammation is recognized as one of the main health problems in hyperlipidemia [44]. Studies conducted on human and animal models have convincingly shown the potential relationship of intestinal inflammation with obesity and IR [45].

It has been established that the activity of myeloperoxidase, a neutrophil infiltration marker, and TLR gene expression are increased in the intestine by HFD. The results of Shi et al., show that intestinal malondialdehyde (MDA) and ROS levels were significantly increased, while the activities of CAT and SOD were reduced by HFD. They also showed that the expression of intestinal genes such as interleukin 10 (IL-10), transforming growth factor β1 and 2 (TGF-β1 and TGF-β2), and IΚβα (the protein product of the NFKBIA gene) were significantly reduced, while the expression of tumor necrosis factor-alpha (TNF-α) and IL-1β genes were increased in fish model fed HFD [46].

Oxidative stress is involved in the pathogenesis of many diseases, including neurodegenerative disorders, IR, T2D, cardiovascular disease, and even cancer [47]. The HFD has been known to be associated with high production of ROS and reactive nitrogen species (RNS) [48]. Previous experiments have shown that HFD increases the expression of unfolded protein response (UPR) signaling molecule Edem1, ERAD chaperone, Grp78, ER chaperone, and sXbp1. These genes are known as markers of endoplasmic reticulum (ER) stress that is related to oxidative stress.

HFD also induces mitochondrial oxidation of free fatty acids and, consequently, elevates ROS production. Increased ROS levels activate the *Bacteroidetes* pathway, causing the release of proinflammatory cytokines such as inducible nitric oxide synthase (iNOS), TNF-α and IFNγ [31]. HFD also increases inflammatory cytokine and monocyte chemoattractant protein-1 (MCP-1) levels [49], where these factors are associated with alterations in gut bacteria, especially *Coprococcus*, *Pseudobutyrivibrio*, and *Dorea* spp., populations [50]. For example, administration of *Bifidobacterium* spp. significantly reduces IL-6 and MCP-1 gene expression in mice [51].

Alteration of gut microbiota or high levels of oxidative stress induced by HFD can increase low-grade inflammation through the TLR and nuclear factor kappa B (NF-κB) pathways [52]. Accordingly, increased intestinal inflammation due to downstream cascades (i.e., NF-κB signaling), eventually causes systemic inflammation and IR [53]. TLR4 activation leads to increased inflammatory responses via c-Jun NH2-terminal kinase (JNK) and IκB kinase complex (IKKβ) and induces IR [54] whereas, IR induced by free fatty acids (FFA) or obesity is significantly reduced in TLR4 knockout animal models, indicating a pivotal role of this receptor in inflammation, metabolic disorders, and IR [55,56].

### 3.5 HFD affects intestinal microbiota

Human and animal model studies have revealed that HFD affects the composition of gut microbiota and leads to high levels of blood lipid, metabolic endotoxemia, and IR [1,3]. In fact, any changes in gut microbiota lead to increasing the pathogenic bacteria, increasing LPS levels, and decreasing the short-chain fatty acids (SCFAs)-producing bacteria [3]. Animal models fed with HFD are commonly used for evaluating the relationship between gut microbiota and diet-induced obesity. In this respect, it has been shown that obesity could not be induced by HF/HSD in germ-free animal models compared with the normal group [3].

HFD can alter the gut microbiota via the reduction of *Bacteroides*, *Bifidobacterium* spp*.,* and *Eubacterium Rectale–Clostridium Coccoides*. A negative correlation between blood lipopolysaccharide levels and *Bifidobacterium* spp., has been reported, and an increase in the *Bifidobacteria* population correlated with the decreased endotoxemia [32].

It has been reported that HFD reduces the counts of beneficial intestinal *Lactobacillus* spp*., Bacteroides-Prevotella* spp., and *Bifidobacterium* spp [57]. Murri et al., have reported that the number of *Clostridium* and *Bacteroides* cells significantly increases and the number of *Lactobacillus* and *Bifidobacterium* cells significantly decreases in diabetic children compared with healthy children [58]. *Bifidobacterium* spp., normalize glucose homeostasis, reduce weight gain and fat mass, and improve glucose-mediated insulin release [59].

A large body of evidence indicates that administration of *Lactobacillus* spp. and *Bifidobacterium* spp. attenuates IR [27]. Le et al., [51] demonstrated that *Bifidobacterium* spp., restores glucose and adiponectin levels and reduce levels of inflammatory markers, and consequently reverses IR in animal models. Simon et al., have reported that administration of *Lactobacillus* normalizes the secretion of incretin and insulin in glucose-tolerant subjects [60]. Moreover, *Lactobacillus* and *Bifidobacterium* increase glucose transporter 4 (GLUT-4), GLP-1 and IL-10 expression in subjects with T2D and obesity, and inhibit lipid accumulation in adipocytes [61].

Recovery of *Bifidobacterium* spp., can associate with reduced inflammation, improved glucose tolerance, and glucose-induced insulin secretion [62]. Lê et al., showed that *Bifidobacteria* populations significantly reduced and L. acidophilus, Lactobacillus, L. rhamnosum, and L. bugaricus significantly increased in the fecal sample of diabetic patients, indicating that the abundance of these bacteria can be known as a microbial biomarker for diabetes [62]. *Bifidobacteria* can decrease the levels of endotoxins by increasing gut barrier integrity promoting a stable environment in the intestine and reducing the translocation of toxins and bacteria. However, increasing the count of gram-negative bacteria can increase intestinal permeability and cause higher lipopolysaccharide level in blood circulation [32]. Barrier permeability induced by gut flora's dysbiosis can cause inflammation and consequently IR [63].

Animal and human studies revealed that the numbers of *Bacteroidetes* are reduced and *Firmicutes* are increased by HFD and obesity [24,33]. The changes in the ratio of *Firmicutes/Bacteroidetes* (F/B ratio) is linked to the prevalence of obesity [3]. Turnbaugh et al., reported that high fat/high sugar diet (HF/HSD) increases the population of the *Erysipelotrichi* and *Bacilli* classes of the *Firmicutes* compared to the control group [64]. The results of Ley et al. experiment revealed that people with obesity displayed a low number of Bacteroidetes compared with control [25]. Furthermore, a significant reduction in the population of lactic acid bacteria, *Enterococcus* and *Bacillus Bifidus* in the intestine of the group fed an HFD was reported. Moreover, *Bacillus fusiformis* showed an increase, and phylum *Bacteroidetes* showed a reducing trend by HFD [33].

It has been reported that the microbiome Shannon index (diversity of gut microbiota) was potentially reduced by HFD [65]. Chen et al., in a cross-sectional study conducted on 2166 subjects, showed that a higher microbiome Shannon index was related to less IR and T2D. They suggested that gut microbiota provide insight into the pathogenesis, etiology, and treatment of T2D [66].

A diet rich in saturated and trans fatty acids can reduce microbiota diversity. In contrast, a diet rich in unsaturated fatty acids, such as omega-3 and omega-6 fatty acids, enhances microbiome diversity and reduces the ratio of Firmicutes/Bacteroides in humans. It has been shown that saturated fats increase the population of gram-negative bacteria, including *Anaerotruncus, Lachnospiraceae, Escherichia, Fusobacterium*, *Eisenbergiella*, and *Tyzzerella.* lipopolysaccharide production by these bacteria induces chronic inflammation and hyperglycemia. Unsaturated fatty acids have been shown to increase the population of butyrate-producing bacteria, including *Eubacterium, Roseburia*, *Bifidobacterium, and Lactobacillus*, along with reductions in *Phascolarctobacterium* and *Veillonella* [67].

### 3.6 Effects of HFD on gut permeability

Animal and human experiments have shown the link between HFD and obesity with elevated endotoxemia, which increases intestinal permeability, reduces intestinal barrier integrity, and consequently enhances translocations of LPS to blood circulation [[68], [69], [70]]. It has been documented that the HFD in animal models changes gut microbiota similar to that of obese subjects [31]. lipopolysaccharide also reduces gut integrity by reducing the expression of tight junction (TJ) proteins, including ZO-1, occluden, and claudin [71].

TJ, along with the mucous layer, has a vital role in the intestinal barrier function. TJs are transmembrane protein strands that link laterally adjacent cells near the epithelium and apical surface. TJs include TJ-associated MARVEL domain-containing proteins (TAMPs), tight junction proteins 1 and 2 (TJP1 and 2), junctional adhesion molecules (JAMs), cingulin, OCLN, and claudins [31].

It has been established that *Lactobacillus* spp., and *Bifidobacterium* spp., (both often reduced in HFD) induce the intestinal gene expression of the TJ proteins cingulin, OCLN, TJP1, and TJP2 [1]. *Lactobacillus fermentum* also increased expression of the GLP-1, GLUT-4, and tight junction proteins (e.g., Zonula occludens-1 (ZO-1)), improving glucose tolerance in animal models [61].

Previous data demonstrated that HFDs reduce gut barrier–promoting microbes, such as *Akkermansia muciniphilia, Clostridiales* spp., *Bacteriodetes* spp., *Bifidobacterium* spp., and *Lactobacillus* spp., while increasing microbes involved in reducing barrier integrity, such as *Desulfovibrio* spp., and *Oscillibacter* spp. These microbiotas modulate intestinal permeability and affect the release of inflammatory cytokines and gut barrier integrity. Furthermore, HFD increases *Desulfovibrio* spp., specifically *Bilophila wadsworthia*, which produces genotoxic H_2_S gas, causing hyperpermeability [1]. It has been reported that the HFD increased the *Oscillibacter* spp., which directly correlates with reduced expression of TJP1 in the intestine [31].

### 3.7 Effects of HFD on SCFA

Intestinal microbiota contributes to digestion, absorption, immune system maintenance, secondary bile acids (BAs) metabolism, catabolism of proteins, degradation of xenobiotics, synthesis of vitamins, and fermentation of plant fibers [72,73]. Products of gut bacterial fermentation such as SCFAs (e.g., propionate, acetate, and butyrate) can affect energy homeostasis [22]. Administration of butyrate precursor drugs (e.g., tributyrin) in animal models has shown protection from IR, liver steatosis, and obesity [22]. SCFAs can regulate the physiological activity of colonic cells, inflammatory processes, appetite, and insulin response [22]. Moreover, the SCFAs regulate the leptin production in adipocytes, and have a central role in human basal metabolism, controlling appetite, GLP-1 and insulin secretion, and glucose homeostasis [71].

Dysregulated concentration of SCFAs has been reported to be involved in the pathogenesis of metabolic disorders and IR [22]. Furthermore, butyrate and propionate have anti-obesogenic action which can induce leptin and anorexigenic hormone synthesis. Whereas, acetate mainly has obesogenic effects inducing ghrelin release, and promoting fat storage [22]. Gut rich in SCFAs-produces bacteria such as *Lachnospiraceae, Bacteroides, Prevotella*, and *Ruminococcaceae* [3].

### 3.8 Mechanism of SCFA

SCFAs play a crucial role in maintaining epithelial barrier integrity, and a decrease in propionate, and butyrate-producing bacteria contributes to reduced intestinal permeability. Butyrate reduces intestinal permeability and inflammation via multiple mechanisms, such as increased TJ protein expression (e.g., zonulin and occluding), mucin production, and suppression of the NF-κB pathway through the stimulation of peroxisome proliferator-activated receptor gamma (PPAR-γ) [31]. Furthermore, acetate also potentially increases epithelial survival and integrity [24]. It has been established that HFD significantly reduces SCFA levels, resulting in reduced intestinal permeability, increased prevalence of pathogenic bacteria, increased release of pro-inflammatory factors, and energy metabolism disorders [3].

As mentioned above, *Lactobacillus* and *Bifidobacterium* were significantly reduced by HFD. These bacteria, the main producers of SCFAs, have been correlated with reduced HbA1c levels in serum. Likewise, reduced absorption of SCFAs has been reported in T2D patients as a result of decreased intestinal barrier integrity induced by dysbiosis [63].

**Fig. 3:**
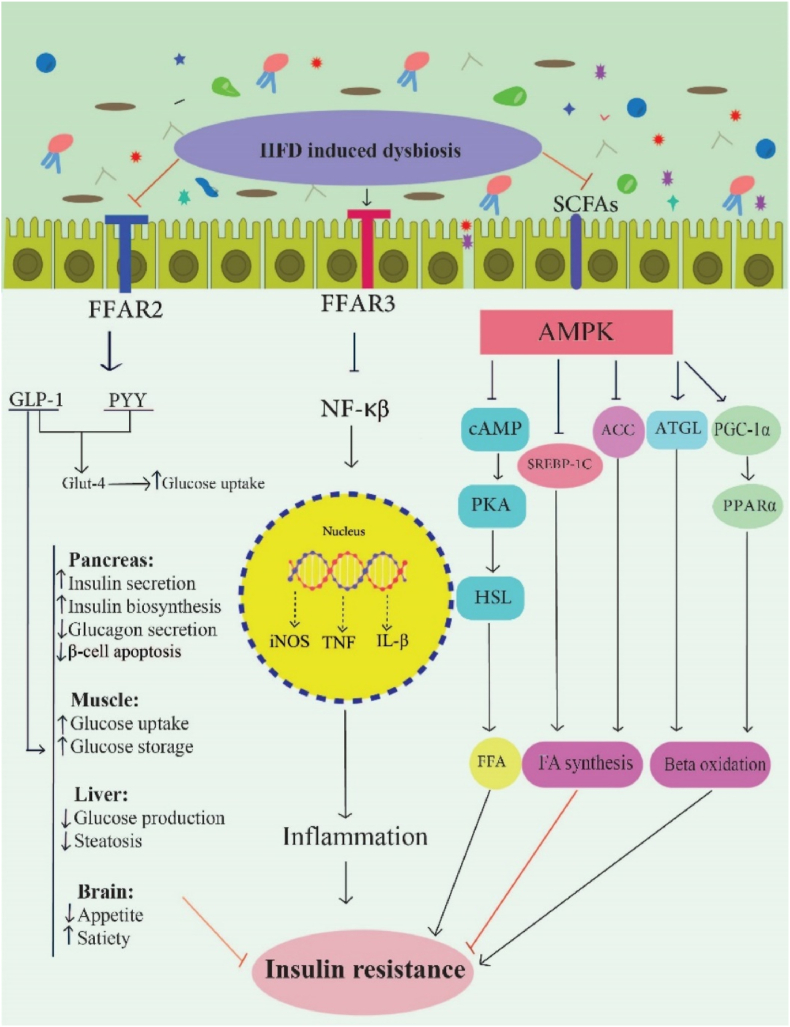
The possible mechanisms of SCFAs regulating metabolism and inflammation [44,71]. SCFAs: short-chain fatty acids; HFD; High fat diet; FFAR2, Free Fatty Acid Receptor 2; FFAR3, Free Fatty Acid Receptor 3; GLP-1, glucagon-like peptide-1; PYY, peptide YY; GLUT4, glucose transporter 4; NF-kB, nuclear factor kappa-B; iNOS, Inducible nitric oxide synthase; IL-1b, Interleukin-1b; TNFa, Tumor necrosis factor α; IR, insulin resistance; AMPK, Adenosine 5′-monophosphate (AMP)-activated protein kinase; PKA, protein kinase A; cAMP, Cyclic adenosine monophosphate; HSL, hormone-sensitive lipase; FFA, free fatty acid; PGC-1a, Peroxisome proliferator-activated receptor gamma coactivator 1a; ATGL, Adipose triglyceride lipase; PPAR, peroxisome proliferator activated receptor; SREBP, sterol regulatory element-binding protein; FA, Fatty acid.

SCFAs interact with G protein-coupled receptors (GPCRs) (Fig. 3), including free fatty acid receptors 2 and 3 (FFAR2, FFAR3), hydroxycarboxylic acid receptor HCA2 (GPR109A), and olfactory receptor 78 (OLFR78), which are involved in improving diabetes-related pathways, such as insulin sensitivity, glucose metabolism, inflammation, and fatty acid oxidation [71].

SCFAs activate the 5′-AMP-activated protein kinase (AMPK), which induces the expression of PPAR, peroxisome proliferator-activated receptor-γ coactivator (PGC)-1α, adipose triglyceride lipase (ATGL), and uncoupling protein (UCP), and inhibits acetyl-CoA carboxylase (ACC). Activation of these proteins promotes energy expenditure and fatty acid oxidation and leads to decreased glucose intolerance and IR [71]. SCFAs also increase intestinal barrier function through the AMPK, reducing gut permeability and preventing the translocation of inflammatory molecules and pathogens into the blood [74].

SCFAs activate FFAR2 (GPR43) and increase the secretion of peptide YY (PYY) and GLP-1 in the intestine, which leads to the increased expression of GLUT-4, and improved glucose uptake. GLP-1 increases insulin secretion and reduces glucagon production in the pancreas, while PYY increases glucose uptake in adipose tissue and muscle. SCFAs also via FFAR2/3 mRNA, increase adiponectin level, elevate expression of genes involved in oxidation of lipids, and reduce markers of lipolysis [71].

SCFAs bind to the FFAR3 (GPR41) and show anti-inflammatory effects by inhibiting the nuclear factor kappa B (NF-κB) pathway and then suppressing the release of TNF-*α*, IL-6, and iNOS. Moreover, SCFAs show anti-inflammatory effects by activating FFAR2 and inhibiting histone deacetylase (HDAC) [44,71]. SCFAs via the GPR109A promote nuclear factor erythroid 2-related factor 2 (NRF2), nuclear import, and induce autophagy, resulting in an anti-inflammatory effect. They also regulate lipid metabolism and insulin sensitivity. Moreover, SCFAs increase mucus production and intestinal barrier integrity [44,71]. HFD can induce dysbiosis by reducing SCFAs and decreasing the activation of FFAR2 and FFAR3. Furthermore, HFD can increase the activation of the TLR pathway. Hence, HFD by disturbance of glucose metabolism and increase of inflammation and lipid biosynthesis can increase the risk of IR (Fig. 3).

SCFA (reduced by HFD) can decrease intestinal inflammation and intestinal permeability through the NF-κB pathway and TJ protein gene expression [24,31]. Alteration in intestinal permeability by HFD is due to the increase in pathogenic bacteria population and reduction of the TJ-related proteins [24]. T helper 17 cells (Th17) are another factor that regulates intestinal barrier integrity. Reduced levels of Th17 were reported in HFD and failure of Th17 response leads to gut permeability and increases lipopolysaccharide translocation in blood [31].

### 3.9 Effects of HFD on endotoxemia

The HFD has direct effects on gut microbiota, which decreases microbial diversity and increases pathogenic microbiota. Change of gut microbiota leads to inflammation (indirect effect), increasing the risk of IR and obesity [75].

In healthy conditions, the physiological level of LPS does not cause negative health effects in the gut. Nevertheless, HFD can increase the production of endotoxemia, which is caused by increased LPS levels in blood circulation by gram-negative bacteria [32]. Previous experiments have documented that LPS levels are increased in obese humans and animal models [76,77]. Although these findings may seem as a paradox, the obese subject showed an increased population of gram-positive bacteria, such as *Firmicutes*, indicating a direct correlation between high LPS levels and increased intestinal permeability [24].

Changes in intestinal integrity by HFD are probably because of decreased expression of tight junction-related components and proteins such as occluding, ZO-1, and claudin, which impedes the endotoxemia and bacterial population from the intestinal lumen to blood circulation. The disturbed tight-junction function increases LPS translocation which is an early factor in the development of inflammation and IR [24]. HFD also increases chylomicron production and accumulation in the intercellular space of the intestine, which can elevate the local pressure, leading to tight junction loosening [31]. In addition to the breakdown of intestinal tight junctions by HFD, the LPS which is produced by pathogenic bacteria, can be bound to chylomicrons in the bloodstream, suggesting that a high-fat diet raises the uptake of these endotoxins [78]. Therefore, HFD by increasing chylomicron production, increases both LPS uptake and translocations [79].

Dysbiosis in HFD-fed animals increases intestinal permeability and consequently translocates the LPS of bacteria into the bloodstream and induces low-grade inflammation which in turn contributes to the pathogenesis of IR [22]. It was demonstrated that infusion of LPS into an animal model on a normal diet can induce an inflammatory response, impaired glucose tolerance, and lead to IR and obesity [24].

It is well known that SCFAs, by suppressing appetite, inducing browning of white adipose tissue, and inhibiting lipogenesis, show a potential anti-obesity effect. It has been shown that SCFAs can suppress de novo lipogenesis by inhibiting acetyl-CoA carboxylase-1(Acc1), the rate-limiting step in de novo fatty acid biosynthesis. Moreover, propionate prevents the TG accumulation by affecting PPARα-responsive genes. Hence, we added this paragraph to the manuscript [80].

The results of a study by Pendyala et al., [81] shows that HFD significantly increased the LPS-containing bacteria in the intestine [33]. Administration of HFD increased the population of *Enterobacteriaceae* in the feces of mice. Moreover, Jeong et al. reported that HFD increased both blood and fecal endotoxin levels and increased the growth of *Enterobacteriaceae* and endotoxin production [31]. An elevated number of LPS-producing bacteria, such as *Desulfovibrionaceae* and *Enterobacteriaceae* which belong to *Proteobacteria*, may be treated as the potential etiology of obesity in HFD-fed animal models [3].

**Fig. 4:**
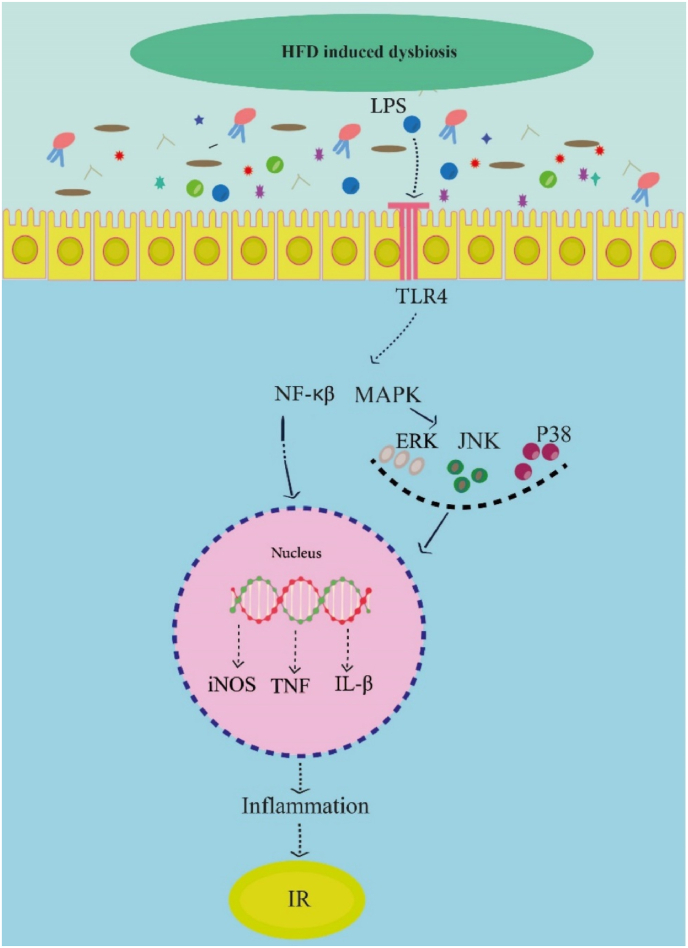
The possible mechanisms by which LPS induce inflammation and insulin resistance [82]. LPS, Lipopolysaccharide; TLR4, Toll-like receptor 4; MAPK, mitogen-activated protein kinase; NF-kB, nuclear factor kappa-B; iNOS, Inducible nitric oxide synthase; IL-1b, Interleukin-1b, TNFa, Tumor necrosis factor α; IR, insulin resistance.

It has been reported that the binding of endotoxin to TLR4 is associated with IR and impaired glucose metabolism (Fig. 4) [82]. Upon TLR-4 activation by endotoxins, transcription of inflammatory factors such as iNOS, TNF-a, IL-6, and IL-1β are increased via MAPK and NF-kB pathways. These inflammatory pathways are potentially increased in diabetic patients, consequently resulting in IR and dysfunction of the pancreatic β-cell [71]. Hence, gut dysbiosis elevates intestinal permeability, causing metabolic endotoxemia, inducing chronic inflammation, by activating TLR4/NF-κB pathways. This condition interferes with insulin signaling, promoting IR(83).

### 3.10 Non-bacterial microbiota in insulin resistance

Although bacteria are well-recognized in the prevalence of IR, the gut also has archaea, viruses, and fungi that are affected by HFD. The roles and importance of archaea, viruses, and fungi in the prevalence of IR are less studied. However, previous studies reported that HFD reduces beneficial fungi (e,g,. Saccharomyces) and increases Candida population that disrupts gut barrier integrity and activates TLR2. HFD also changed Archaea in the intestine. In this respect, HFD increases *Methanobrevibacter smithii*, which raises adiposity and energy harvest [[83], [84], [85]].

### 3.11 Herbal medicine and gut microbiota

Medicinal plants have been used for the prevention and treatment of many diseases for thousands of years. When herbal plants enter the gut, they interact with microbiota, regulate the gut microbiota, maintain intestinal homeostasis, and limit inflammatory response. Bioactive fractions of medicinal plants can induce microorganisms to secrete certain endogenous substances responsible for increasing immune surveillance and barrier integrity [86].

Medicinal plants have an essential role in maintaining health by improving insulin sensitivity, inflammation, and oxidative stress. They can prevent IR by possible hypolipidemic, hypoglycemic, and anti-inflammatory effects [[87], [88], [89], [90]]. On the one hand, herbal medicines with antioxidant and anti-inflammatory effects can affect intestinal function and structure and regulate the composition of microbiota [91]. It has been established that many medicinal plants significantly inhibit the growth of pathogenic bacteria such as *Escherichia*, *Verrucomicrobia*, *Staphylicus*, and *Clostridium*, while increasing the population of beneficial bacteria such as *lactobacilli*, *Bifidobacteria*, *Blautia*, and *Akkermansia* [86].

### 3.12 Prevention strategy

Administration of probiotics is an exceptional option to help replace the intestinal microbiota. Probiotics can normalize intestinal hormones and modulate oxidative stress [92]. For example, lactic acid bacteria can reduce pro-inflammatory cytokines. Nevertheless, one key issue for long-term use of the Western diet is that the use of probiotics provides only a limited number of intestinal bacterial cells. Previous experiments have shown that the administration of probiotics in diabetic animals normalizes blood glucose, increases insulin sensitivity, and improves pancreas function and structure [92]. We have also shown that probiotics normalized brain function and structure in diabetic animals [93]. The strain, delivery methods, and dosage are essential factors affecting the efficacy of probiotics in controlling insulin sensitivity and blood glucose. The optimal dose for humans is unclear, and host factors (e.g., genetics, gastric acid resistance, gut microbiota, and diet) can affect probiotic treatment. Furthermore, not all probiotic strains show glucose-lowering effects.

High fiber-containing foods and supplements, such as bilberry, erythritol, guar gum, pinto beans, inulin, coarse cereal mixture, betaine, baicalin, nobiletin, phlorizin, tea extract, green banana, jamun fruit extract, flaxseed fiber, euglena, anthocyanins, can increase the SCFA levels and SCFA-producing bacteria in the intestine [94].

Since the present study is carried out as a Narrative review rather than Systemic or Met-analysis and based on the limitation of our study, the outstanding questions that should be addressed by future studies include (i) the differential effects of omega-3-, omega-6-, and omega-9-rich high fat diets on the gut microbiota, (ii) the effects of HFD on the awakening of sterile inflammation (activation of NLRP3 inflammasome), (iii) formulation of probiotics with optimized strain, delivery method, and effective dosage, (iv) the effect of disrupted gut microbiota on the adipose tissue metabolism, (v) the molecular mechanism of contribution of HFD in gut-brain axis and cognitive function, and (vi) introducing a more effective therapeutic strategy to prevent HFD-induced IR, oxidative stress, and dysbiosis.

## 4 Conclusion

This review article revealed that changes in gut microbiota in HFD-fed models were associated with IR. Our study shows that the relative abundance of pathogenic bacteria is significantly elevated after an HFD, which can increase the risk of inflammation and IR. Furthermore, this paper also showed that SCFAs, endotoxins, and other metabolites can be considered commonalities and features of gut microbiota metabolites in IR. Dysbiosis induced by HFD can mainly affect SCFAs to induce IR through the activation of the NF-κB and inhibition of PPAR-γ pathways. However, the interactions of SCFAs with various receptors and signaling pathways (e.g., AMPK, PPAR-γ) are complex, and more studies are needed to clarify these mechanisms.

## CRediT authorship contribution statement

**Ebrahim Abbasi:** Writing – original draft, Visualization, Validation, Software, Methodology, Investigation, Funding acquisition, Formal analysis. **Iraj Khodadadi:** Validation, Supervision, Software, Resources, Methodology, Investigation, Funding acquisition, Conceptualization.

## Availability of data and materials

The data are presented in the manuscript.

## Funding

None.

## Declaration of competing interest

The authors have no conflicts of interest to declare.
